# Digitalisierung und regionaler Wissenstransfer: Interdependenzen und Herausforderungen

**DOI:** 10.1007/s00548-023-00844-3

**Published:** 2023-03-31

**Authors:** Philipp Bäumle, Daniel Hirschmann, Daniel Feser, Simon J. Winkler-Portmann, Kilian Bizer

**Affiliations:** 1 Lehrstuhl für Wirtschaftspolitik und Mittelstandsforschung, Georg-August-Universität Göttingen, Platz der Göttinger Sieben 3, 37073 Göttingen, Deutschland; 2 Fachbereich Gesellschaftswissenschaften, Hochschule Darmstadt, Haardtring 100, 64295 Darmstadt, Deutschland

**Keywords:** Wirtschaftsförderung, Innovationsförderung, Regionale Wissenstransfersysteme, Wissensintermediäre, Regionale Entwicklung, Knowledge transfer, Digitalization, Regional knowledge dynamics, Knowledge intermediaries, Regional development, I29, O32, O39

## Abstract

Die Digitalisierung von Unternehmen und der Wissenstransfer (WT) zwischen akademischen und nichtakademischen Akteuren sind zentral für regionale Innovationsstrategien. Dennoch ist weder klar, wie der WT zur Digitalisierung beitragen kann, noch wie digitale Arbeitsweisen den WT unterstützen können. Um beide Fragestellungen näher zu beleuchten, untersuchen wir sechs regionale Wissenstransfersysteme in Deutschland. Die Analyse zeigt, dass Maßnahmen des WT auf unterschiedliche Weise zu Digitalisierungsprozessen regionaler Akteure beitragen und sich die Akteure des WT vielfach digitaler Arbeitsweisen bedienen. Innerhalb regionaler Wissenstransfersysteme werden sowohl Informationen zu digitalisierungsrelevanten Themen vermittelt und in Netzwerken vertieft als auch konkrete Digitalisierungsprojekte unterstützt. Digitale Tools und Arbeitsweisen spielen eine wichtige Rolle, einerseits für die Durchführung entsprechender Veranstaltungsformate und andererseits für die Zusammenarbeit zwischen Organisationen. Die Analyse zeigt jedoch auch, dass die Möglichkeiten der Unterstützung durch WT begrenzt sind und auch hinsichtlich der Nutzung digitaler Instrumente verschiedene Herausforderungen bestehen. Letztere konzentrieren sich auf die Auswahl und Umsetzung bestehender Lösungen, die von wissenschaftlicher Seite nur begrenzt sinnvoll unterstützt werden können. Themen von größerem wissenschaftlichem Interesse finden nur bei wenigen, bereits stark digitalisierten Unternehmen intensivere Beachtung. Darüber hinaus gestaltet es sich schwierig, vertrauensbasierte Netzwerkbeziehungen über digitale Kanäle zu initiieren und für Kooperationen zwischen Organisationen nutzbar zu machen.

## Abstract (en)

Digitalization and knowledge transfer represent central aspects of most regional innovation strategies. Nonetheless, we know little about the interdependencies between them. We know neither how knowledge transfer can contribute to digitalization nor how digital instruments can support knowledge transfer. To shed more light on these issues, we analyzed six regional knowledge transfer systems in Germany. The analysis reveals that knowledge transfer contributes to firm digitalization in several ways and that supporters of knowledge transfer often apply digital instruments. Within regional knowledge transfer systems, intermediaries disseminate relevant knowledge, establish specific networks, and support particular digitalization efforts. Digital tools play an important role in processing these activities as well as in cooperation within knowledge transfer systems. However, it is also clear that the possibilities of knowledge transfer in digitalization are limited and that several challenges remain in utilizing digital instruments in knowledge transfer. Challenges in digitalization often consist of selecting and implementing digital solutions, which cannot always be supported by academia. Innovative academic topics have been taken up by only a few digital pioneers. Further, it remains challenging to initiate and establish trust-based network relationships and to cross administrative borders via digital channels.

## Digitalisierung und Wissenstransfer als standortspezifische Herausforderungen regionaler Innovationspolitik

Die Digitalisierung stellt eine der zentralen Herausforderungen für das wirtschaftliche und gesellschaftliche Zusammenleben dar, mit denen sich Unternehmen und Politik konfrontiert sehen. Gerade für Geschäftsführung und Mitarbeiterschaft kleiner und mittlerer Unternehmen (KMU) gestalten sich die Entwicklung und Adaption von Strategien zur Digitalisierung eigener Prozesse und Geschäftsmodelle herausfordernd (Demary et al. 2016; Öz 2019). Mit der digitalen Transformation sind weitgehende Veränderungen von Berufsfeldern und Arbeitsmärkten (Arntz et al. 2020) sowie Anforderungen an Beschäftigte und Arbeitgeber*innen verbunden (Weber 2017), die über die Ebene von Einzelunternehmen hinausgehen. Auch die regionale Ebene ist relevant für Digitalisierungsprozesse (Zika et al. 2018).

Der Zugang zu externem Wissen stellt in wissensbasierten ökonomischen Strukturen einen wichtigen Bestimmungsfaktor der Wettbewerbs- und Innovationsfähigkeit von Unternehmen und Standorten dar. Politische und gesellschaftliche Erwartungen gegenüber wissenschaftlichen Einrichtungen führen dazu, dass sowohl Universitäten im Rahmen ihrer „third mission“ als auch öffentliche Akteure den wechselseitigen WT zwischen Wissenschaft, Wirtschaft und Gesellschaft aktiv fördern (Czarnitzki et al. 2001; Bercovitz und Feldman 2006).^1^ Universitäten sind zunehmend gefordert, sich von „Elfenbeintürmen“, zu gesellschaftlich engagierten Akteuren zu entwickeln, um den rekursiven Transfer produzierten Wissens zwischen Wissenschaft, Wirtschaft und Gesellschaft zu ermöglichen (Breznitz und Feldman 2012). Der Aufbau regionaler Netzwerkstrukturen, die KMU den Zugang zu akademischem Wissen und damit die Teilhabe an Innovationsprozessen ermöglichen, ist zentraler Bestandteil innovationspolitischer Strategien (Back und Fürst 2011), beispielsweise im Kontext clusterpolitischer Ansätze (Wissenschaftsrat 2007). Akteure der Wirtschafts- und Innovationsförderung haben in den vergangenen Jahren sowohl WT als auch Digitalisierung als zentrale Arbeitsfelder erkannt. In diesem Sinne verstehen politische Strategien WT zunehmend als Mechanismus zur Förderung von Digitalisierung (Wissenschaftsrat 2016; Sachverständigenrat 2020).

Hinsichtlich zweier zentraler Aspekte besteht Forschungsbedarf: Erstens wirft die steigende Relevanz der Digitalisierung die Frage auf, inwiefern regionale WT-Strukturen die Veränderungen in Unternehmen unterstützen. Zweitens stellt Digitalisierung neben steigendem Wettbewerbsdruck zwischen Wissenschaftseinrichtungen und sich ändernden gesellschaftlichen Erwartungen eine der zentralen Herausforderungen für WT-Formate dar (Wissenschaftsrat 2016), sodass Diskussionen darüber aufkommen, wie WT-Akteure selbst von der Adaption digitaler Kommunikationskanäle und Werkzeuge profitieren können (Ebers 2021; Terstriep und Rabadijeva 2021). Dieser Beitrag nimmt zwei verschiedene Perspektiven ein, um einen Beitrag an der Schnittstelle zwischen WT und Digitalisierung zu leisten. Einerseits zeigt der Beitrag auf, wie WT-Systeme Digitalisierungsprozesse in KMU fördern, und beleuchtet damit die nach außen gerichtete Perspektive der analysierten WT-Systeme. Andererseits adressiert der Beitrag die interne Perspektive der WT-Akteure, indem er Herausforderungen identifiziert, die bei der Digitalisierung eigener Aktivitäten und Strukturen auftreten. Gemäß dem explorativen Charakter des Forschungsthemas und der genutzten Datengrundlage zielt der Beitrag darauf ab, die Interdependenzen zwischen WT und Digitalisierung als Gegenstand zukünftiger innovationspolitischer Forschungsvorhaben und Instrumente zu positionieren. Spezifische Detailergebnisse zu präsentieren liegt deswegen nicht im Fokus der Analyse.

Auf Basis einer Analyse von sechs regionalen WT-Systemen adressiert der Beitrag folgende Forschungsfragen:

1. *Wie tragen regionale Wissenstransfersysteme zu Digitalisierungsprozessen in KMU bei?*
2. *Welche Herausforderungen treten bei der Verwendung digitaler Wissenstransferformate auf?*

## Quellstudien und Methodik

Zur Beantwortung der Forschungsfragen verwenden wir einen Multi-Fallstudienansatz zur Analyse von sechs WT-Systemen in den Regionen Augsburg, Darmstadt, Eberswalde, Göttingen, Osnabrück und Hannover. Hierbei untersuchen wir 56 Expert*inneninterviews mit an WT beteiligten Stakeholdern, insbesondere Wirtschaftsförderungseinrichtungen (18), Transferstellen von Wissenschaftseinrichtungen (24), Kammern (9) sowie Unternehmen bzw. Unternehmensverbänden (5). Die Untersuchung basiert auf zwei Forschungsprojekten zu WT-Strukturen in Deutschland. Der Fokus der Forschungsprojekte lag auf dem Potenzial regionaler WT-Systeme für Nachhaltige Entwicklung (NE) (Studie 1, siehe Bäumle et al. 2022) und dem Zusammenwirken verschiedener Akteure der Innovationsförderung (Studie 2, siehe Bäumle und Bizer 2022). Studie 1 zielte auf die Erforschung der Rolle von NE in WT-Systemen und basierte auf je zwei Regionen mit und ohne explizitem Fokus auf NE. Studie 2 adressierte Voraussetzungen und Effekte der strategischen Zusammenarbeit zwischen verschiedenen WT-Akteuren und berücksichtigte drei Regionen.

**Abb. 1:**
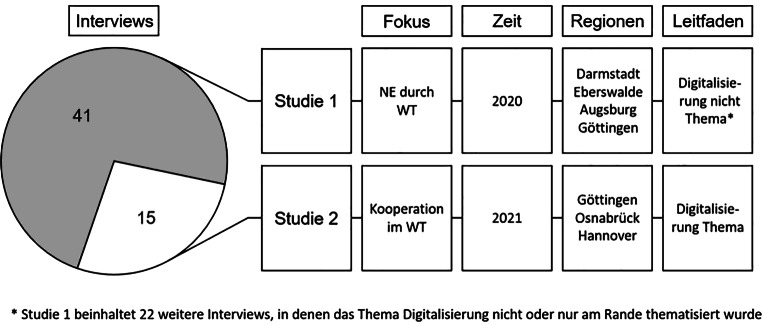
Eckpunkte der genutzten Erhebungen. *Quelle*: Eigene Darstellung

Abb. 1 gibt einen Überblick über die genutzten Interviews. Entsprechend der Neuartigkeit unseres Forschungsinteresses, analysieren wir die Interviews induktiv und explorativ, um die maßgeblichen Schnittstellen zwischen Digitalisierung und WT zu identifizieren. Dabei ist zu beachten, dass alle Interviews nach Beginn der COVID-19-Pandemie bzw. dem Beschluss der damit verbundenen Einschränkungen durchgeführt wurden.

## Ergebnisse

Den Forschungsfragen folgend, gliedern sich die Ergebnisse in zwei Teile. Zunächst stehen die Ergebnisse zur Unterstützung von Digitalisierungsprozessen in KMU durch regionale WT-Systeme im Fokus. Darauf folgen die Herausforderungen, die mit der Digitalisierung von WT-Strukturen selbst einhergehen.

### Unterstützung der Digitalisierung regionaler KMU

Viele KMU haben einen erheblichen Orientierungsbedarf und schrecken häufig vor dem Ausmaß der Veränderungen zurück, die Digitalisierung auslösen kann: Sensibilisierung und Peer-Learning sowie Unterstützung bei der Umsetzung sind deshalb von großer Bedeutung.

#### Sensibilisierung und Weiterentwicklung des Problembewusstseins

**Abb. 2:**
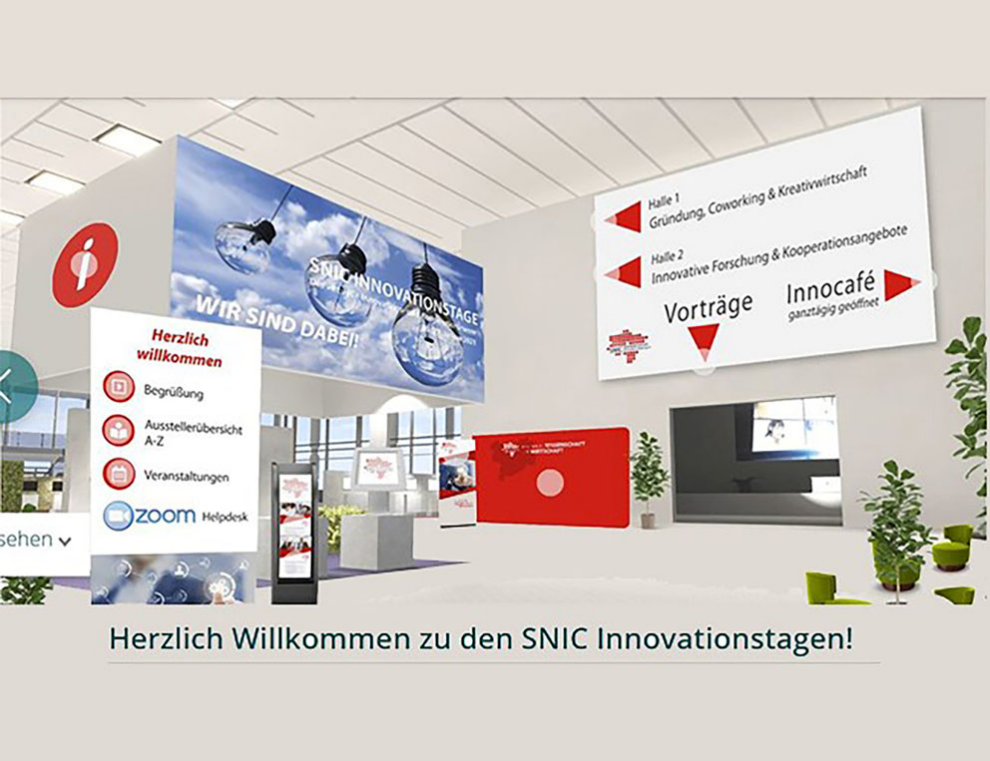
Besucher*innenoberfläche bei der digitalen Kooperations- und Innovationsmesse des SüdNiedersachsenInnovationsCampus. *Quelle:* SNIC (2021)

Der erste Schritt bei Digitalisierungsprozessen innerhalb von Unternehmensstrukturen besteht darin, ein Problembewusstsein unter den Verantwortlichen zu schaffen. Dazu bedarf es gezielter Maßnahmen zur Sensibilisierung und der Möglichkeit, Bedarfe, Ziele und Anwendung der Digitalisierung zu kommunizieren. In allen betrachteten WT-Systemen wurden kooperative Veranstaltungsformate zur Informationsvermittlung entwickelt und durchgeführt. Daran sind insbesondere universitäre Transferstellen und Wirtschaftsförderungseinrichtungen beteiligt. Zudem sind Akteure aus der Wissenschaft eingebunden, indem sie demonstrieren, welche Möglichkeiten aus aktuellen Forschungsvorhaben entstehen. In diesem Zusammenhang beschreiben die Befragten unterschiedliche Formen und Zuschnitte von Veranstaltungen. Auf der einen Seite ziehen Veranstaltungen zu Grundlagenthemen und mit breit gefächerten Zielgruppen viele Teilnehmende an. Beispielhaft ist die virtuelle Innovations- und Kooperationsmesse des SüdniedersachsenInnovationsCampus zu nennen, die in den vergangenen beiden Jahren jeweils dreistellige Teilnehmendenzahlen zu verzeichnen hatte und zuletzt das Thema „Nachhaltigkeit und digitale Transformation“ in den Fokus rückte (Abb. 2). Dies ergänzen Veranstaltungsformate mit stärkerem thematischem Fokus und branchenspezifischen Zielgruppen. Wiederholt wurde der Mehrwert von Good-Practice-Beispielen betont, in deren Rahmen Unternehmen anderen Unternehmen – entweder am eigenen Standort oder in wissenschaftlichen Einrichtungen – Einblicke in gelungene Digitalisierungsprozesse gewähren.

#### Peer-Learning und Unterstützung der Netzwerkbildung

Aufbauend auf der über die Veranstaltungsformate geschaffenen Sensibilisierung wurden innerhalb der analysierten WT-Systeme regelmäßige Dialogveranstaltungen zu Potenzialen der Digitalisierung etabliert. In diesen Netzwerken können Vertreter*innen aus KMU, Wissenschaft, Verwaltung und Gesellschaft verschiedene Perspektiven kennenlernen und Wissen und Erfahrungen austauschen. So werden innerhalb der Netzwerke Peer-Learning-Prozesse induziert.

Innerhalb der Wissenstransfersysteme sind Netzwerke an den Schnittstellen zwischen Unternehmensnetzwerken und Akteuren aus der Wissenschaft entstanden und durch Akteure des WT aufrechterhalten worden. In diesem Kontext sind neben dem Austausch in regionalen Netzwerken auch nationale und internationale Verflechtungen von Bedeutung. Besonders die Vernetzung mit bestehenden Innovationsschwerpunkten und -netzwerken ist wichtig, um Wissensvorsprünge weiter zu stärken und digitale Innovationen zu ermöglichen.

#### Unterstützung der Implementation

**Abb. 3:**
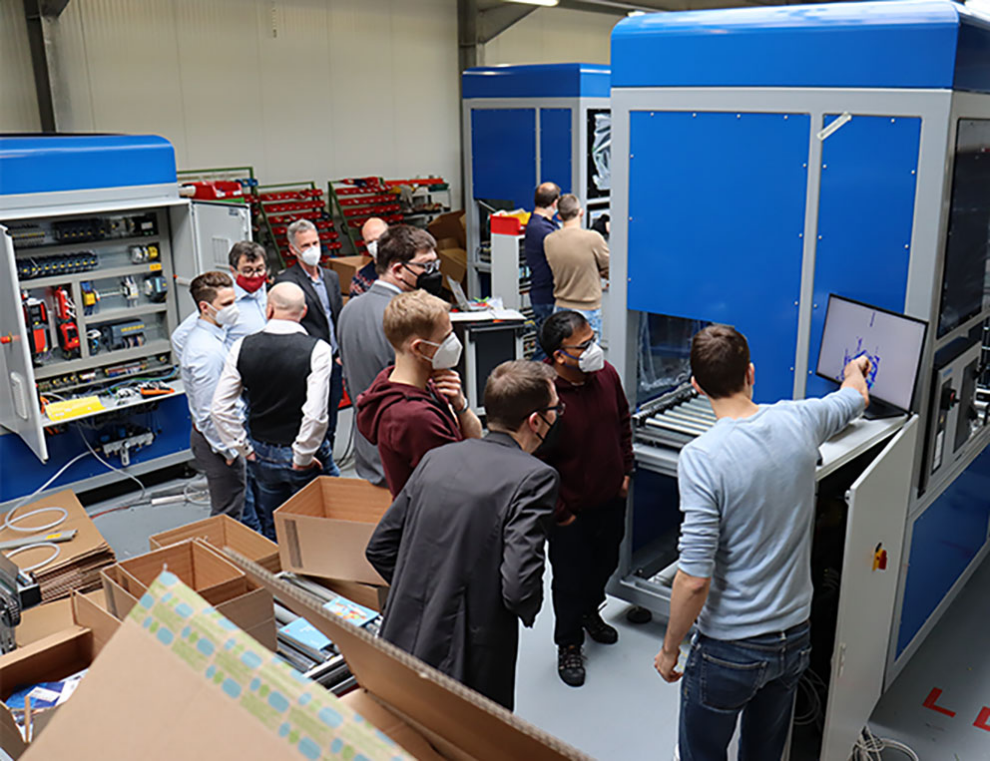
Informationsveranstaltung in einem KMU, das ein kooperatives Innovationsprojekt umsetzt. *Quelle: *SNIC (2022)

Aus themenspezifischen Innovationsnetzwerken entwickeln sich Kooperationen, in deren Rahmen WT-Akteure aktiv zur Implementation digitaler Technologien in Unternehmen beitragen. Vorrangige Zielgruppe dieser Formate sind KMU, die beabsichtigen, identifizierte Digitalisierungspotenziale umzusetzen (s. Abb. 3). Die Interviews thematisieren Partnerschaften zwischen Hochschulen und Akteuren der kommunalen Wirtschaftsförderung, in denen letztere Beratungsangebote durch Wissenschaftler*innen fördern und so den direkten WT gewährleisten. In Einzelfällen berichten die Interviewpartner*innen auch von der Einrichtung zusätzlicher Personalstellen in hochschuleigenen Transferstrukturen mit dem Ziel, Wissenstransferprojekte zur Förderung der Digitalisierung im Mittelstand zu initiieren und zu begleiten. Hierbei handelt es sich jedoch um projekt- und zeitgebundene Stellen. Darüber hinaus werden Studierende in Digitalisierungsprozesse eingebunden: Ihre digitalen Kompetenzen fließen über Seminare und Abschlussarbeiten in Problemlösungsprozesse ein. Zugleich werden so die digitalen Kompetenzen der Studierenden als zukünftige Fachkräfte gefördert.

Die Interviews verdeutlichen jedoch, dass sich viele digitalisierungsspezifische Herausforderungen nicht durch originäre Maßnahmen des WT adressieren lassen. Unternehmen geht es häufig darum, aus vielen Möglichkeiten die am besten geeignete auszuwählen und die notwendige Akzeptanz und Qualifikation der Belegschaft zu gewährleisten oder bestehende digitale Lösungen an eigene Bedarfe anzupassen. Die Neuentwicklung von Instrumenten, die durch Wissenschaftler*innen unterstützt werden könnte, erscheint nur in Einzelfällen zielführend. Dies liegt nicht zuletzt in dem niedrigen Digitalisierungslevel vieler KMU begründet. So berichten die Befragten, dass digitale Zukunftsthemen wie Cloud-Technologien und künstliche Intelligenz (noch) nicht Bestandteil der alltäglichen Arbeit sind.

### Digitalisierung eigener Aktivitäten und Strukturen

Für die WT-Akteure verändern sich durch Digitalisierung die eigene Arbeitsweise und regionale Kooperationsstrukturen. Digitale Kommunikationskanäle erhöhen die geografische Reichweite der Aktivitäten und erlauben es, größere Zielgruppen zu adressieren bzw. spezialisierte Angebote zu gestalten und dafür die kritische Masse zu generieren. Die Integration neuer Instrumente und Formate fiel den meisten Befragten – nicht zuletzt durch den pandemieinduzierten Anpassungsdruck – relativ leicht. Die Befragten betonen den für sie selbst erleichterten Zugang zu digitalen Informationsangeboten von Kooperationspartnern. Dies vereinfacht die individuelle Fortbildung und den Austausch von Erfahrungen. Jedoch benennen die Befragten in diesem Zusammenhang auch verschiedene Herausforderungen.

#### Bevorstehender Generationenwechsel

WT-Akteure stehen vor einem Generationenwechsel, der sich auf die Organisationsstrukturen des WT auswirken wird. Jüngere Befragte nehmen Digitalisierungsprozesse grundsätzlich als positiv und erfolgreich wahr, betonen in diesem Zusammenhang aber intergenerationale Diskrepanzen hinsichtlich der Bereitschaft, digitale Arbeitsweisen zu akzeptieren und zu adaptieren. Neue Formen der Aus- und Weiterbildung sowie der persönlichen Sozialisation sorgen dafür, dass jüngere Beschäftigte den Aufbau neuer Strukturen anstreben. Älteren Beschäftigten fällt es schwerer, die gewohnten, auf physische Präsenz ausgerichteten Arbeitsweisen weiterzuentwickeln. Das Gelingen von Digitalisierungsprozessen hängt demnach auch von einer erfolgreichen Moderation des Generationenwechsels und gegenseitiger Lernprozesse zwischen Beschäftigtengruppen mit unterschiedlichen Ausbildungshintergründen ab.

#### Inhaltlich-methodische Herausforderungen

Ungeachtet der Relevanz und der Potenziale der Digitalisierung für den WT, betonen die Interviewpartner*innen die Grenzen digitaler Kommunikation und Zusammenarbeit. Demzufolge ist die Ansprache zusätzlicher Akteure bzw. die Verbreitung von Informationen ohne größere Einschnitte auch über digitale Kanäle möglich. Zusätzlich bieten digitale Tools die Möglichkeit, personengebundenes Wissen und Netzwerke bei Personalwechseln innerhalb der eigenen Organisation weiterzugeben. Jedoch gestaltet sich die Netzwerkarbeit über digitale Kanäle schwieriger. Im Zentrum vieler WT-Aktivitäten steht, Vertrauensverhältnisse sowie kreative Kollaboration aufzubauen, die sich nicht oder nur eingeschränkt über physische Distanz induzieren lassen. Die Befragten berichten beispielsweise von neuen Mitgliedern in Innovationsnetzwerken, zu denen sie noch keinen persönlichen Kontakt und daher kein belastbares Vertrauensverhältnis aufbauen konnten. Zwar beobachten die Befragten Fortschritte bei der Entwicklung neuer Softwarelösungen, die diese Problemstellungen adressieren, den persönlichen Kontakt können diese jedoch nur bedingt substituieren. Zukünftig bedarf es den Befragten zufolge eines Modus, der Vorteile und Potenziale beider Herangehensweisen nutzbar macht. Vielfach besteht die Motivation, einzelne, seit Pandemiebeginn erfolgreich erprobte Formate auch weiterhin im virtuellen Raum zu verankern bzw. physische Treffen oder Veranstaltungen zu reduzieren.

#### Infrastrukturelle und regulatorische Herausforderungen

Obschon die Interviewpartner*innen von der proaktiven Implementation verschiedener infrastruktureller und organisatorischer digitaler Anpassungen berichten, betonen sie auch, dass die digitale Zusammenarbeit über Organisationsgrenzen hinweg schwierig sein kann. Zum einen sind unterschiedliche digitale Lösungen etabliert, die technisch nicht immer auf eine organisationsübergreifende Zusammenarbeit ausgelegt sind. Zum anderen führen der teilweise hohe Grad an Sensibilität der anfallenden Daten und die daraus resultierenden datenschutzrechtlichen Einschränkungen zu Schwierigkeiten bei Kooperationen. Eine gemeinsame, regional – im Idealfall gar überregional – einheitliche Informationsinfrastruktur könnte die Zusammenarbeit untereinander als auch mit den Zielgruppen vereinfachen. In den Interviews beschriebene Versuche scheiterten an technologischen und regulatorischen Problemen. Daraus ergibt sich ein Bedarf an digitalen Tools, welche die Kollaboration über Organisationsgrenzen hinweg und die Aufbereitung regionsspezifischer Daten und Entwicklungen rechtssicher ermöglichen.

## Fazit und standortpolitische Implikationen

Die Analyse zeigt, dass Akteure und Mechanismen des WT die Digitalisierung regionaler KMU auf unterschiedliche Arten unterstützen. Vor allem Veranstaltungsformate und Netzwerke sind wichtig, um ein Problembewusstsein zu schaffen und darauf aufbauend Lernprozesse anzustoßen. Diese Erkenntnisse unterstreichen die Bedeutung standortspezifischer Perspektiven für die Entwicklung funktionierender Fördermaßnahmen zur Digitalisierung. Hochschulische Wissenstransfereinrichtungen können kommunale und private Akteure bei der Konzeption und Ausrichtung niedrigschwelliger Veranstaltungs- und Beratungsangebote zur Steigerung des Problembewusstseins regionaler Akteure unterstützen. Konkrete Bedarfe und Vorhaben von KMU im Kontext der Digitalisierung lassen sich jedoch nicht in allen Fällen durch WT fördern. Nichtsdestotrotz ist die Informations- und Netzwerkarbeit innerhalb regionaler WT-Systeme Grundlage und Ausgangspunkt für eine intensivere Zusammenarbeit, um Digitalisierungsherausforderungen zu überwinden.

**Abb. 4:**
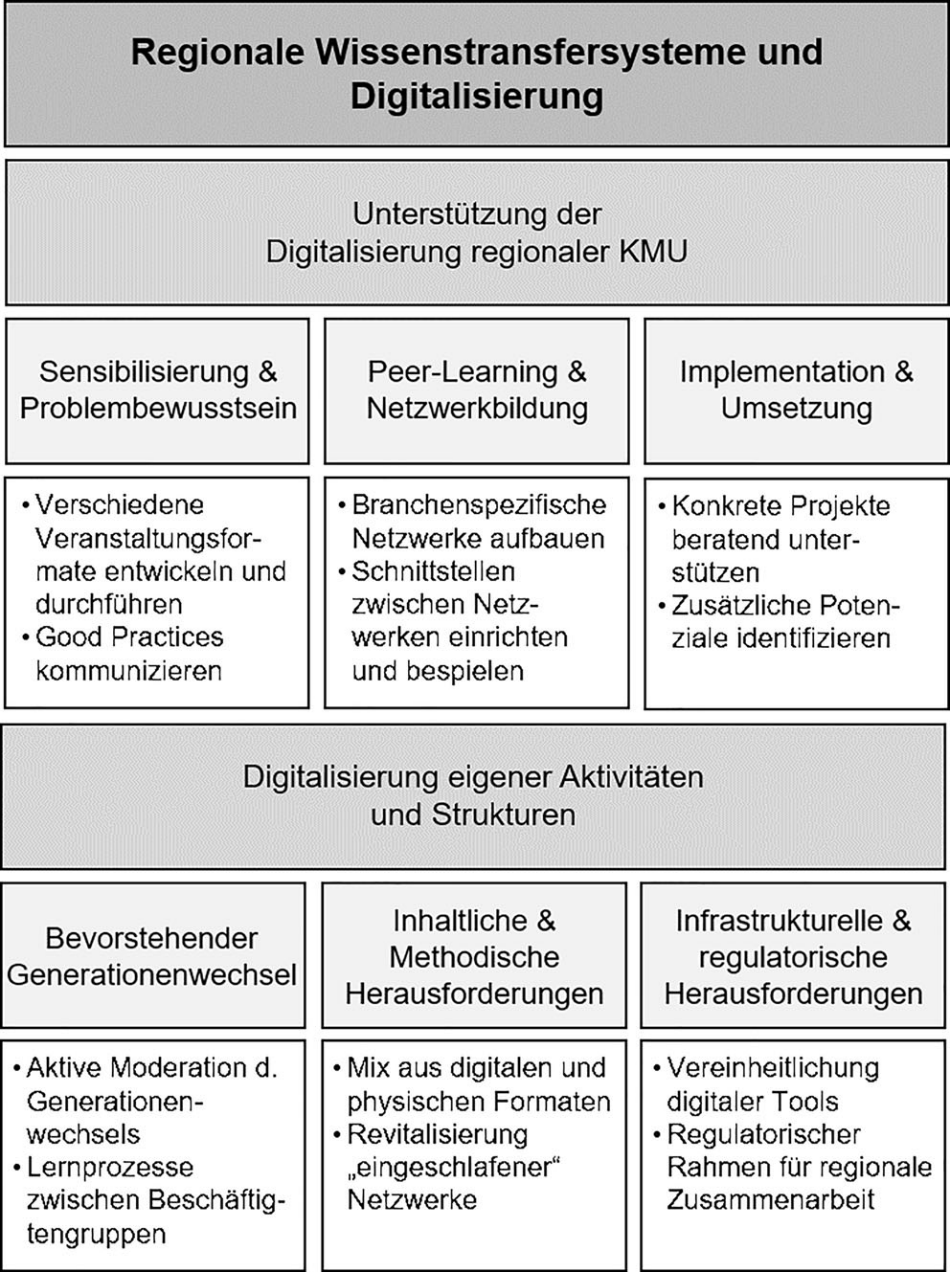
Regionale Wissenstransfersysteme und Digitalisierung: Herausforderungen und Lösungsansätze. *Quelle:* Eigene Darstellung

Die Befragungen der WT-Akteure zeigen auch die Grenzen der Digitalisierung der eigenen Arbeit. Einerseits lassen sich Aktivitäten mit dem Ziel der Verbreitung oder dem Austausch von Informationen in digitale Räume verlagern. Andererseits lassen sich Formate mit dem Ziel vertrauensbasierter Netzwerkbildung oder der Verknüpfung einander unbekannter Akteure nur in begrenztem Maße digital durchführen. Hinzu kommen Herausforderungen hinsichtlich der Akzeptanz innerhalb des WT sowie datenschutzrechtlicher und infrastruktureller Probleme (Abb. 4).

Die Potenziale des WT für die Digitalisierung sollten auf regionaler Ebene aufgegriffen werden. Dafür ist standortspezifisch herauszuarbeiten, zu welchen Anstrengungen im Bereich Digitalisierung die Aktivitäten des WT beitragen können und zu welchen nicht. Insbesondere die Anknüpfung an regional verfügbares Wissen ist für die Diffusion digitaler Technologien entscheidend. Weiterhin gilt es zu analysieren, ob die Zielsetzungen einer WT-Aktivität ein digitales Format zulassen. Zu diesem Zweck ist die Weiterbildung von Mitarbeiter*innen und der Abbau infrastruktureller Barrieren zentral.

Abschließend offenbart dieser Beitrag zwei maßgebliche Forschungsbedarfe: Erstens bedarf es detaillierter Fallstudien, die auf standortspezifische Kontexte eingehen und die Interdependenzen zwischen WT und Digitalisierung in unterschiedlich organisierten WT-Systemen analysieren. Zweitens bedarf es eines besseren Verständnisses, wie sich die verschiedenen Digitalisierungsaspekte auf die Wirksamkeit des WT auswirken.

## Footnotes

1 Der Beitrag legt ein breites Verständnis des Begriffs Wissenstransfer zugrunde, das alle Aktivitäten umfasst, die nötig sind, um Wissen im Kontext einer anderen Organisation in Wert zu setzen (vgl. hierzu Wit-de Vries et al. 2019, S. 1238).
